# Dynamic Electronic Tracking and Escalation to reduce Critical care Transfers (DETECT): the protocol for a stepped wedge mixed method study to explore the clinical effectiveness, clinical utility and cost-effectiveness of an electronic physiological surveillance system for use in children

**DOI:** 10.1186/s12887-019-1745-7

**Published:** 2019-10-17

**Authors:** Gerri Sefton, Bernie Carter, Steven Lane, Matthew Peak, Ceu Mateus, Jen Preston, Fulya Mehta, Bruce Hollingsworth, Roger Killen, Enitan D. Carrol

**Affiliations:** 1 Alder Hey Children’s NHS Foundation Trust, Liverpool, L12 2AP UK; 2 Edge Hill University, Ormskirk, L39 4QP UK; 3 University of Liverpool, Liverpool, L69 3BX UK; 4 NIHR Alder Hey Clinical Research Facility, Alder Hey Children’s NHS Foundation Trust, Liverpool, L12 2AP UK; 5 Lancaster University , Lancashire, LA1 4YG UK; 6 University of Exeter, Exeter, EX4 4PU UK

**Keywords:** Deterioration, Sepsis, VitalPAC®, Paediatric early warning (PEWS), E-obs, Careflow vitals and connect

## Abstract

**Background:**

Active monitoring of hospitalised adults, using handheld electronic physiological surveillance systems, is associated with reduced in-patient mortality in the UK. Potential also exists to improve the recognition and response to deterioration in hospitalised children. However, the clinical effectiveness, the clinical utility, and the cost-effectiveness of this technology to reduce paediatric critical deterioration, have not been evaluated in an NHS environment.

**Method:**

This is a non-randomised stepped-wedge prospective mixed methods study. Participants will be in-patients under the age of 18 years, at a tertiary children’s hospital. Day-case, neonatal surgery and Paediatric Intensive Care Unit (PICU) patients will be excluded.

The intervention is the implementation of Careflow Vitals and Connect (System C) to document vital signs and sepsis screening. The underpinning age-specific Paediatric Early Warning Score (PEWS) risk model calculates PEWS and provides associated clinical decision support. Real-time data of deterioration risk are immediately visible to the entire clinical team to optimise situation awareness, the chronology of the escalation and response are captured with automated reporting of the organisational safety profile.

Baseline data will be collected prospectively for 1 year preceding the intervention. Following a 3 month implementation period, 1 year of post-intervention data will be collected.

The primary outcome is unplanned transfers to critical care (HDU and/or PICU). The secondary outcomes are critical deterioration events (CDE), the timeliness of critical care transfer, the critical care interventions required, critical care length of stay and outcome.

The clinical effectiveness will be measured by prevalence of CDE per 1000 hospital admissions and per 1000 non-PICU bed days.

Observation, field notes, e-surveys and focused interviews will be used to establish the clinical utility of the technology to healthcare professionals and the acceptability to in-patient families. The cost-effectiveness will be analysed using Health Related Group costs per day for the critical care and hospital stay for up to 90 days post CDE.

**Discussion:**

If the technology is effective at reducing CDE in hospitalised children it could be deployed widely, to reduce morbidity and mortality, and associated costs.

**Trial registration:**

Current Controlled Trials ISRCTN61279068, date of registration 03.06.19, retrospectively registered.

## Background

All-cause child mortality in the UK has not maintained improvement, in line with other European countries. The Confidential Enquiry into Maternal and Child Health deaths (CEMACH) ‘Why children die”, found that 25% of in-hospital deaths had identifiable failures in direct care and potentially avoidable factors in 43% [1, 2]. The factors included failure to recognise and respond effectively to deterioration. Paediatric Early Warning Scores (PEWS) and systems have been advocated as a mechanism to assist healthcare staff to identify deterioration so that care might be augmented to improve outcome. However, the evidence base underpinning their use in clinical practice is limited [1]. In the UK, despite reported use of PEWS by 85% of hospital treating children [3], there are more than 4000 emergency transfers to Paediatric Intensive Care Units per year from in-patient wards [4], suggesting that those patients deteriorated in hospital. The progression of deterioration to become critical, requiring admission to a high dependency unit (HDU) or the PICU is known to increase the length of hospital stay and the risk of mortality [5–7]. Evolving evidence suggests that PEWS may assist in reducing the severity of illness and the critical interventions required by patients who require unplanned emergency transfers to critical care [8, 9].

To date, PEWS research has been hampered by the use of paper-based charting of vital signs. Achieving implementation fidelity of PEWS systems have been challenging, with incomplete monitoring of the specific vital signs that contribute to PEWS reported. Additionally, there has been poor documentation of PEWS escalation, the timeliness of response and the associated clinical outcomes, which makes it difficult to assess if PEWS have been implemented as intended. In contrast, active monitoring of adult patients in hospital using an electronic physiological surveillance system has been in place for more than 10 years [10]. This technology has been associated with the standardisation of vital signs monitoring and has demonstrated a significant reduction in deaths at two large University hospitals in the UK [11]. That work contributed to the development of a National Early Warning Score (NEWS), a single risk model for identifying signs of early deterioration in adults [12–14].

### Why this study is necessary and the expected contribution to the field

The risk model developed for adults is unsuitable for use in children for a number of reasons;

1. age-associated variation in physiological signs including heart rate, respiratory rate and blood pressure, from birth to adulthood, mean that a single PEWS risk model would be inadequate to flag deterioration across the age ranges [15–18].
2. the ability to physiologically compensate for serious illness is limited in children, providing a shorter window of opportunity for staff or parents to recognise the signs and to respond quickly, to avoid the progression of deterioration to become critical [19, 20].

To date, there is no technology similar to that used in adults, which has been robustly tested for use for children in hospital, for screening physiological signs using an underpinning age-specific PEWS risk model. The most extensive randomised study of PEWS in an international context, the EPOCH study, did not demonstrate a reduction in mortality associated with the use of PEWS [1]. A lower observed event rate for mortality occurred in both arms, of the intervention and comparison sites. The observed mortality was lower than the data used for the computation of the sample size estimate (2007 data from fourteen hospitals). The study also highlighted the challenge in achieving complete data capture of the required vital signs which contribute to PEWS when paper-based documentation was used. Less than 5% of all the recorded data reviewed [1] were complete for recording of the seven components which had contributed to the validation of the bedside PEWS.

The development of electronic physiological surveillance systems suitable for use in a paediatric population is complex and expensive, potentially deterring industry from developing age-specific products. To address this deficit, The Learning Clinic (now System C) collaborated with this research team to develop technology underpinned by scientific evidence. Proof-of-concept testing of the VitalPAC Paediatric prototype occurred at this hospital site, alongside existing practice. When compared to paper charts, VitalPAC Paediatric demonstrated improved the accuracy of vital sign documentation; 98.5% Vs 85.6% *p* < 0.02; PEWS calculation; 94.6% Vs 55.7% *p* < 0.02 and reduced the documentation time; 68 Vs 98 s *p* < 0.002 [21].

Sepsis is a major cause of deterioration, therefore addition of a paediatric sepsis [22, 23] clinical prompt, including laboratory and clinical data was tested. This demonstrated 71% sensitivity and 81% specificity when two or more components were present, suggesting it could be an effective tool to prompt nurses and doctors to consider sepsis earlier. The staff using the technology perceived that this mode of documentation improved safety and increased team situation awareness.

This study is the whole hospital evaluation of electronic physiological surveillance technology to provide pro-active monitoring for paediatric in-patients.

## Methods/design

SMART technology, self-monitoring and reporting technologies, such as electronic physiological surveillance systems have the potential to provide an end-to-end solution for recognising and responding to serious illness or deterioration in children. The study aims to:

- Standardise the process for monitoring vital signs for children in hospital
- Improve the detection of early signs of clinical deterioration, including sepsis
- Provide real-time data to the entire clinical team to improve team situation awareness about deteriorating patients
- Use clinical decision support to prompt the pro-active management of deterioration, and early stabilisation of patient, reducing the progression of deterioration to become critical
- Reduce emergency transfers to critical care following deterioration in hospital
- To prompt timely transfer of deteriorating patients to critical care, who were non-responders to first-line stabilisation interventions

### Study design

This is a non-randomised stepped-wedge prospective mixed methods study exploring the clinical effectiveness, clinical utility and cost-effectiveness of an electronic physiological surveillance system to provide active monitoring of paediatric patients and to screen for early signs of serious illness, deterioration or sepsis.

#### Population and setting

The participants will be ward in-patients, aged less than 18 years, at a tertiary children’s hospital in the United Kingdom. The study site has 240 in-patient beds, a large emergency department, a 24-bed PICU, a 19-bed HDU and a 4-bed long-term ventilation Unit (LTVU).

The patient profile will include a mix of general paediatric medical and surgical patients and tertiary specialties including oncology, cardiology/cardiac surgery, neurology/neurosurgery, burns, respiratory, renal and orthopaedics. Day-case patients, patients in the neonatal surgical unit or in the PICU will be excluded.

The hospital does not have a Medical Emergency Team or a Critical Care Outreach Team. Patient deterioration is managed by the core clinical team. During the out-of-hours period, patients are managed by the on-call team. If a call to the resuscitation team is made, the responding team comprise of a senior nurse, senior paediatric registrars, resuscitation training officers (day-time only Monday to Friday), the Paediatric Intensive Care registrar and/or an anaesthetist.

#### The intervention

The intervention is an electronic physiological surveillance system suitable for use in children in hospital. This is composed of health IT software Careflow Vitals (previously known as VitalPAC) configured with an underpinning age-specific PEWS risk model, which runs wirelessly on Apple hand-held devices. The technology is used to manually document vital signs to screen for early signs of serious illness or deterioration, including sepsis. The software prompts an active monitoring process by standardising the complete documentation of vital signs and clinical assessments, at a frequency linked to the child’s PEWS. The completed PEWS are categorised as low, moderate, high or critical risk for deterioration, with associated decision support prompted for each. When a suspicion of sepsis has been documented, a complete sepsis assessment is required to stratify the clinical concern regarding sepsis using the NICE sepsis pathway [22], this prompts targeted care delivery and captures the time critical sepsis bundle compliance.

Careflow Vitals is used in conjunction with Careflow Connect; a secure encrypted communication system for professionals to provide real-time data to the entire clinical team on hand-held devices. Automated alerts about high or critical PEWS categorisation are sent to the Nurse in Charge of the ward and to the responsible clinical team. The chronology of escalation, response and associated actions are captured.

There will be phased implementation of the technology ward by ward, with concurrent assessment of the implementation fidelity. Weekly organisational safety reports will be fed back to wards, clinical teams and managers. These will collate reporting of the completeness and frequency of assessment, active management of patients with high or critical PEWS or new sepsis concerns, and the timeliness of emergency transfers to critical care.

### Comparison

Baseline data will be collected prospectively for 1 year preceding the intervention, with patient monitoring of vital signs and sepsis screening recorded in the Electronic Patient Record (Meditech 6). The hospital standard for the monitoring of vital signs for children in hospital, is set out in a clinical policy, together with the expected escalation and response processes. All of the component parts required for the calculation of a core PEWS (respiratory rate, effort of breathing, oxygen saturation (SpO2), oxygen delivery (FiO2), heart rate, capillary refill time and nurse concern) are mandated within the EPR each time vital signs are recorded. Temperature is also recorded but it does not contribute to the PEWS. For new admissions, PEWS score 3 or more or clinical concern, requires that an extended PEWS is undertaken. This incorporates additional vital signs alongside the core PEWS; which include blood pressure, AVPU (rapid neurological assessment of responsiveness; Alert-Verbal-Pain-Unresponsive) and parental concern. The process for communication for concern of deterioration is through the traditional bleep process.

Following a 3 month implementation period, 1 year of post-intervention data will be collected.

#### Outcomes

The primary outcome is unplanned transfers to critical care, either to HDU and/or PICU.

The secondary outcomes are critical deterioration events [24], the patients who had unplanned transfers from in-patient wards to critical care (HDU and/or PICU) and required organ support in the following 12 h. The CDE prevalence per 1000 hospital admissions and per 1000 non-ICU bed days will be reported. The timeliness of critical care transfer will be reported using the Children’s Resuscitation Intensity Scale (CRIS) [25]. The severity of illness at unplanned transfer to PICU; the Prognostic index of Mortality (PIM3 [26], the requirement for critical care interventions (mechanical ventilation, non-invasive ventilation, inotropes and/or dialysis), length of critical care stay and outcome. We will report the sepsis screening, suspicion of sepsis, clinical confirmation of ‘treat as sepsis’, the NICE sepsis bundle compliance and associated outcome.

The activation of the resuscitation team for cardiac arrests, respiratory arrests, or peri-arrest situations in the in-patient wards (excluding PICU) will be reported. All-cause mortality will be reported for the wards included in the study. The overall critical care activity; total admissions, bed days, length of stay, refused admissions (internal/external) and cancellations of planned major surgery due to lack of critical care capacity, will be reported.

#### Sample size

The study will collect data from 240 in-patient beds. Based on existing hospital data, over 12 months 300–350 emergency transfers to HDU and/or PICU from in-patient wards would be expected. Previously published work from this centre [8] suggested that a robustly implemented process for proactively recognising and responding to deterioration in hospital, would contribute to a 30% reduction in total bed days following a CDE, with a median reduction of two critical care bed days per CDE. This is projected to be sufficient for the analysis planned.

The projected 80–100% bed occupancy in 240 beds is expected to yield a minimum of 6 sets of vital signs/per bed/per day, which is expected to yield a minimum of 630 K–780 K observation sets per year.

#### Data analysis

The data analysis is broken down into the four main work-packages in the study.

1. The quantitative analysis of the clinical effectiveness at reducing critical deterioration, will report the prevalence of CDEs per 1000 hospital admissions and per 1000 non PICU bed days to compare the baseline and intervention arm of the study.
  The predictive ability of the age-specific PEWS risk model, will be reported using the sensitivity, specificity, positive/negative predictive values Modelling of the physiological values will be used to determine evidence-based thresholds for vital signs and logistic regression models will be developed to determine the weighting of components of the PEWS risk model to improve the performance at improving early detection of signs of deterioration. The study will use statistical process control techniques and will explore the feasibility of using stochastic control methods to track the condition of patients over-time to develop a longitudinal component (trend changes in vital signs), within the risk model.
2. The qualitative analysis will focus on the clinical utility of the technology to assist in the recognition and response to deterioration in children in hospital. It will also aim to evaluate the acceptability to children and their families of being monitored in this way. The three core methods which will be used are ethnographically driven observation and conversational interviews, short e-surveys, and focused interviews. Qualitative analysis will use an interpretive, reflexive, conceptual thematic analytical approach [27, 28] the integration of descriptive codes and themes into robust concepts via Morse’s four cognitive analytical processes [29].
3. The analysis of proactive sepsis screening for all patients will describe the prevalence of pre-existing risk factors for sepsis, the cases where sepsis was suspected, the subset where the clinician made a decision to treat as sepsis (blood cultures and intravenous antibiotics), NICE sepsis bundle compliance and associated outcome. Multi-variate modelling will be also be used to determine the components of the sepsis assessment which would contribute to improved discrimination of evolving sepsis.
4. The cost-effectiveness of the technology will be analysed using Health Related Group (HRG) costs per day for the critical care and hospital stay following a CDE for up to 90 days, pre and post intervention. The societal perspective will be used and the time horizon is 1 year. For medical direct costs, patient level hospital costs will be computed by using hospital costs and published tariffs where appropriate. Indirect costs include work absenteeism in the parents of children who had an in-hospital deterioration, and productivity losses resulting from the sequelae of critical illness.

## Discussion

The type of active monitoring has been in place for adults in hospital for more than 10 years [12–14]. It was evaluated in two large university hospitals in an NHS setting in the UK and was associated with a reduction in in-hospital mortality [11]. The ability to record real-time vital signs data on mobile devices which are instantly visible to the entire clinical team, together with automated critical alerts, tracked monitoring, escalation and response provides metrics for hospitals to review their patient safety at ward, clinical teams and organisational levels. Although mortality is much rarer for children in hospital, it is envisaged that the use of active monitoring could reduce the progression of deterioration to become critical.

Critical Deterioration Events [30] were associated with a 13-fold increased risk of mortality in hospital. It is a clearly defined outcome measure for deterioration which has been reported in two paediatric studies [1, 30]. There is widespread acceptance that the threshold for admission to critical care varies world-wide, therefore, it is useful to subdivide the admissions to be explicit about CDEs, although, at present, this categorization is not routinely undertaken. However, there is evidence about unplanned admissions to PICU that does demonstrate a reduction in the median length of critical care stay by 2 days following the implementation of a paper-based PEWS [8].

Despite this, it has been challenging to sustain those improvements, over time, not least because of the human resource required to ensure that organisational process are followed. The use of self-monitoring and reporting technologies (SMART) can provide frequent feedback to healthcare professionals and managers to drive culture change, and can provide assurance to healthcare regulators of safe processes for patients. Up to now paper-based monitoring of vital signs documentation and organisational processes has meant that it is difficult to ensure adequate implementation fidelity of PEWS.

If the technology is clinically effective at reducing CDE in hospitalised children, it could be deployed widely, to reduce the morbidity and mortality associated with critical deterioration.

## Data Availability

A minimum dataset associated with the study outcomes, will be made available through linked documents uploaded alongside any future publications.

## Abbreviations

CDE: Critical Deterioration Events
CRIS: Children’s Resuscitation Intensity Scale
HDU: High Dependency Unit
NiHR: National Institute for Health Research
PICU: Paediatric Intensive Care Unit
PIM3: Paediatric Index of Mortality, version 3
